# Macrophage Migration Inhibitory Factor: Critical Role in Obesity, Insulin Resistance, and Associated Comorbidities

**DOI:** 10.1155/2010/610479

**Published:** 2010-02-09

**Authors:** Robert Kleemann, Richard Bucala

**Affiliations:** ^1^Department of Biosciences, Gaubius Laboratory, TNO-Quality of Life, Zernikedreef 9, 2301 CE Leiden, The Netherlands; ^2^Department of Medicine and Pathology, School of Medicine, Yale University, New Haven, CT 06510, USA

## Abstract

Obesity is associated with insulin resistance, disturbed glucose homeostasis, low grade inflammation, and comorbidities such as type 2 diabetes and cardiovascular disease. The cytokine macrophage migration inhibitory factor (MIF) is an ubiquitously expressed protein that plays a crucial role in many inflammatory and autoimmune disorders. Increasing evidence suggests that MIF also controls metabolic and inflammatory processes underlying the development of metabolic pathologies associated with obesity. This is a comprehensive summary of our current knowledge on the role of MIF in obesity and obesity-associated comorbidities, based on human clinical data as well as animal models of disease.

## 1. Introduction

The incidence of obesity is increasing in many populations in the world and in some regions now affects more than 30% of adults [1]. Increased adipose tissue mass, especially in the abdominal region, is associated with insulin resistance and disturbed glucose homeostasis (dysmetabolic state), and comorbidities such as type 2 diabetes (T2D) and cardiovascular disease [2, 3]. Visceral adiposity is considered to be a marker of the dysmetabolic state, reflecting the inability of subcutaneous adipose tissue to act as a protective metabolic sink for the clearance and storage of dietary triglycerides. Recent advances in our understanding of adipose tissue biology have provided evidence for a more causative role of obesity in the etiology of its comorbidities. Increased fat mass is associated with increased macrophage infiltration, increased release of cytokines, adipokines, and free fatty acids (FFAs) from adipocytes and/or activated macrophages, and local insulin resistance [4]. The release of cytokines/adipokines and FFA into the circulation allows their transport to the liver, heart, aorta, and skeletal muscle, often promoting reduced insulin sensitivity and local inflammation in these organs [5]. Tissue-specific insulin resistance is thought to further enhance a proinflammatory condition because insulin itself can exert antilipolytic and anti-inflammatory effects. On the systemic level, this proinflammatory condition is reflected by elevated levels of cardiometabolic risk factors including C-reactive protein (CRP), fibrinogen and serum amyloid A (SAA), and by circulating mononuclear cells that exhibit a proinflammatory status. 

Macrophage migration inhibitory factor (MIF) is an innate cytokine involved in many inflammatory and autoimmune disorders; many of these pathologies are associated with obesity including cardiovascular [6] and kidney disease [7]. MIF is widely expressed in numerous types of tissue and it regulates acute inflammatory as well as adaptive immune reactions [8, 9]. Increasing evidence suggests that MIF also controls metabolic and inflammatory processes underlying the development of metabolic disorders such as glucose homeostasis during periods of stress and macrophage infiltration into adipose tissue. This survey summarizes our current knowledge on the role of MIF in obesity-associated comorbidities, based on clinical and research data.

## 2. Human Studies

### 2.1. MIF and Obesity

Obesity and specifically the enlargement of the abdominal adipose depots are considered major risk factors for the development of insulin resistance, T2D, and diabetic complications. Several lines of clinical evidence support a relationship between MIF and obesity (Table 1). Dandona et al. reported a correlation between serum MIF levels and the body mass index (BMI) [10]. Obese subjects with an average BMI of 37.5 ± 4.9 kg/m^2^ had a significant higher fasting MIF concentration (2.8 ± 2.0 ng/mL) than healthy lean control subjects (BMI 22.6 ± 3.4 kg/m^2^; 1.2 ± 0.6 ng/mL) [10]. MIF mRNA expression in mononuclear cells (MNCs) was also significantly greater (by 60%) in the obese and related to plasma FFA concentrations and BMI but not to plasma MIF concentrations or HOMA-index. The authors showed that 6 weeks of metformin treatment (obese subjects only) lowered MIF plasma levels in the absence of an effect on plasma glucose, insulin, and FFA. After withdrawal of the drug, MIF levels returned to their initial value indicating a metformin-specific effect. Other studies confirmed elevated plasma MIF levels in the obese compared to lean subjects [11, 12] as well as increased MIF mRNA expression in MNC [11]. In line with these observations, severely obese but otherwise healthy subjects (BMI 43.0 ± 8.6 kg/m^2^) participating in a diet and physical activity-based weight management program showed decreased plasma MIF levels (39% decrease) after weight loss of 14.4 kg [13]. In another weight loss program (4.4 kg weight loss), a 67% decrease in circulating MIF was reported [14]. In contrast to these studies however, morbid obese subjects (BMI 46.7 ± 5.8 kg/m^2^) displayed relatively low baseline plasma MIF levels (about 0.2 ± 0.4 ng/mL) [15]. After gastric restrictive surgery, the BMI decreased markedly (33 ± 4.8 BMI kg/m^2^) in these patients while MIF concentrations remained stable and low for the first 6 months. Then, following weight loss, MIF levels began to increase and reached the levels of lean individuals (0.71 ± 0.58 ng/mL) within 12 weeks after surgery. 

The results of the above studies evaluating the relationship between obesity and MIF are not uniform and any causal relationship between obesity and MIF levels remains to be established. Factors that may contribute to the large variability between the studies include differences in gender (plasma MIF levels are higher in males [16]), the use of hormone replacement therapy (HRP; woman with HRP show 2-3 fold higher plasma MIF levels [13]), circadian rhythm [17], and differences in *MIF* promoter genotypes causing differences in promoter activity. Functional *MIF* promoter alleles which include a variable nucleotide tandem repeat (−794 CAT*T*_5–8_) [18] and associated single nucleotide polymorphisms (SNP) have been identified [19, 20] and recent studies have linked the differences in MIF serum levels between male and female subjects to specific SNPs [16, 21]. 

### 2.2. Studies in Diabetic Patients

Recent epidemiological data provide support for a role for MIF in the development of IR and T2D in humans (Table 2). Compared to normal healthy control subjects, patients with T2D displayed significantly elevated serum MIF levels [24]. MIF was not correlated with plasma glucose, HbA1c, and diabetes duration. The American Pima Indians, which have been intensively studied because of their high incidence of T2D diabetes also show elevated circulating levels of MIF [25]. Herder and coworkers reported a significant increase of circulating MIF, CRP, and interleukin-6 (IL-6) in subjects with impaired glucose tolerance (IGT; *n* = 242) or T2D (*n* = 236) compared to normoglycemic controls (*n* = 244) (KORA S4 study; participants aged 55–74 years) [26]. The strong positive association between systemic concentrations of MIF and IGT and T2D was independent of the other immune mediators. In contrast to CRP and IL-6, there was a highly significant increase in MIF concentrations in the T2D group compared with the IGT group suggesting that elevations of systemic MIF concentrations precede the onset of T2D. 

Additional support for a causal role of MIF in the etiology of T2D comes from a population-based study comparing the effect of four SNPs of MIF (rs755622, rs2070766, rs2070767, and rs1007888) on serum concentrations and the risk of T2D (MONICA/KORA Augsburg Study) [16]. The C allele of SNP rs1007888 was associated with increased circulating MIF independent of age, sex, BMI, blood pressure, TC/HDL cholesterol, CRP, IL-6, smoking status, physical activity, time of blood draw. Female subjects with *MIF* genotype rs1007888CC had an increased risk of T2D [16]. The association between MIF levels and incident T2D was significantly higher in obese women compared with nonobese ones. 

Different results were obtained in the Finnish Diabetes Prevention Study (*n* = 522; 40–65 years old) which tested lifestyle intervention in overweight or obese men and women with impaired glucose tolerance (IGT) and BMI > 25 kg/m^2^ [27]. MIF was not associated with the risk of T2D in the untreated control group of this study. In the intervention group, subjects with high MIF and low RANTES levels had a lower risk of T2D (sexes were not analyzed separately in this study). 

Elevated levels of MIF or its cell surface receptor (CD74) were found in patients with diabetic complications including diabetic nephropathy [24, 28], diabetic retinopathy [24, 29, 30], and diabetic foot syndrome [31] further supporting a role of MIF in the comorbidities of obesity. A recently published transcripitonal profiling study of 364 biopsies obtained before and after hyperinsulinemeic-euglycemic clamp showed significant overepxression of the MIF receptor. Moreover, thiazolidoinedione treatment was associated with a significant downregulation of this gene [32]. Clear evidence for a direct link between obesity and insulin resistance may be deduced from a very recent study in the obese showing that MIF mRNA expression in subcutaneous adipose tissue is positively associated with adipocytes diameter and negatively associated with peripheral and hepatic insulin action [23]. Importantly, freshly isolated mature adipocytes from subcutaneous, omental, and mammary depots can release MIF at rates up to 10,000 pg/mL × 24 hours, and MIF production of the adipose depots is positively correlated with donor BMI [22]. 

The above epidemiological and clinical studies suggest an important link between MIF, obesity, the dysregulation of glucose metabolism, and the development of insulin resistance and T2D, but it remains unclear whether such abnormalities in MIF and MIF receptor expression are epiphenomena or casual factors. In the past few years, studies in experimental models of disease have provided some confirmatory data indicating a mechanistic role for MIF in the comorbidities of obesity.

## 3. Animal Studies

While a role for MIF could be demonstrated in various settings of experimental type I diabetes [33–35], the involvement of MIF in the pathogenesis of insulin resistance/T2D has hardly been explored. Animal studies employing experimental setting relevant for insulin resistance/T2D can be subdivided in (1) studies investigating the role of MIF in glucose metabolism and (2) studies elucidating the role MIF in chronic WAT inflammation which are discussed separately.

### 3.1. Impact of MIF on Glucose Metabolism

First evidence for a role for MIF in glucose metabolism was suggested by the immunohistochemical identification of MIF within the insulin-containing granules of the islets of Langerhans [36]. The MIF content of pancreatic islets was increased by high glucose concentrations, while immunoneutralization of MIF in a perifusion system of Langerhans islets reduced the first and second phases of glucose-induced insulin secretion by 39% and 31%, respectively. MIF addition to pancreatic islets also augmented the glucose-induced insulin secretion in response. A progressive decrease in MIF secretion or sensitivity within the insulin-secreting cells thus may be postulated to contribute to *β*-cell dysfunction, and an elevation in systemic MIF in T2D may represent a physiologic mechanism to overcome this defect [36]. 

The development of a systemic inflammatory response during microbial infection or tissue invasion frequently leads to a catabolic state. Transient hyperglycemia and insulin resistance typically occur first, but this may be followed by a persistent state of lactate production and metabolic acidosis, glycogen depletion, and hypoglycemia. Circulating cytokines such as tumor necrosis factor (TNF) have long been considered to provoke these metabolic abnormalities, which unresolved, lead to protein wasting and cachexia. In murine studies the catabolic effect of TNF on muscle was found to be mediated by MIF [37], as the administration of an anti-MIF antibody was found to abolish the TNF-induced hypoglycemia and the increase in muscle F2, 6 BP levels, which is a powerful allosteric regulator of glycolysis and lactate production. Anti-MIF also prevented these effects in TNF-knockout mice administered bacterial endotoxin, thus confirming the intrinsic contribution of MIF to these inflammation-induced metabolic changes [37]. 

These findings were further extended once MIF-KO mice became available. Hyperinsulinmeiceuglycemic clamp studies in endotoxemic or TNF injected MIF-KO mice showed these mice to exhibit normal blood glucose and lactate responses [38]. MIF-KO mice also had increased glucose uptake into white adipose tissue (WAT). Of note, TNF had been shown previously to induce MIF production from cultured adipocytes [39], and the intracellular content of MIF mRNA and protein also has been shown to be regulated by the intracellular glucose level [40]. Within adipose tissue, MIF inhibited insulin signal transduction [38]. MIF decreased the tyrosine phosphorylation of insuln receptor substrate-1 (IRS-1) and its association with the p85, and it inhibited the insulin-induced phosphorylation of AKT, which is known to provide signals for the synthesis of new glucose transporters. Overall, these studies support the concept that MIF plays an important role in the regulation of systemic glucose metabolism during infection or tissue invasion. Moreover, they suggest that the previously described action of TNF in insulin resistance is in fact mediated by the downstream, autocrine/paracrine action of MIF. 

Finally, a recent study has identified a potentially critical role for MIF in the cardiomyocyte response to ischemia [41]. MIF was found to be released in the ischaemic heart and stimulate the activation of AMPK, which is an enzyme that senses the cellular energy state and affects diverse pathways to increase cellular ATP production and limit energy consumption. Notably, human fibroblasts with a low-expression MIF allele have diminished MIF release and AMPK activation during hypoxia, suggesting that the predetermination of *MIF* genotype might predict risk in patients with coronary artery disease.

### 3.2. Impact of MIF on Chronic WAT Inflammation

We recently showed that genetic deletion of MIF reduces systemic inflammation (SAA and fibrinogen) and protects mice against the development of glucose intolerance and insulin resistance [42]. Development of disease was followed over time (period of 52 weeks) in the LDL receptor deficient mouse model of combined insulin resistance and atherosclerosis. MIF deficiency improved the insulin sensitivity of WAT as quantified by PI3-kinase activity and AKT phosphorylation assays, and there was no corresponding effect in liver or muscle in this model. MIF deficiency did not affect adipose mass or obesity, but the adipocytes of MIF deficient mice however were found to be smaller in size, beginning at a young age (12–18 weeks) [42]. 

An important event in the pathogenesis of insulin resistance of WAT is the infiltration of macrophages causing low-grade adipose inflammation [43] that appears to develop as a consequence of aging and/or high fat dietary stress [44]. MIF-deficiency in the low density lipoprotein receptor (LDLR) knock-out mouse model reduced macrophage infiltration into WAT leading to fewer of the crown-like, histologic structures that are typically seen in insulin resistant adipose tissues. The effect of MIF deficiency may be related to the observed reduced expression of intracellular adhesion molecule-1 (ICAM-1) and CD44 in WAT, that is, two proteins that mediate monocyte/macrophage recruitment. An MIF-dependent influence on adhesion molecule expression and of macrophage tissue infiltration has been reported in the context of cardiovascular disease (in absence of obesity) [45–47]. 

Besides controlling local tissue-specific inflammation, MIF is implicated in controlling systemic inflammation, including the expression of risk factors of T2D. MIF affects the constitutive and interleukin-1 (IL-1)-induced expression of SAA as well as the expression of IL-6 and human CRP as demonstrated in human CRP transgenic mice [42]. MIF-expressing mice also display higher levels of fibrinogen [42], which is a marker of the prothrombotic status and risk factor for T2D. The stimulating effect of MIF on the expression of T2D risk factors (e.g., CRP) appears to reside within the protein's conformationally sensitive catalytic domain(s) [48] because protein mutants of MIF which lack the intrinsic oxidoreductase or tautomerase activity [49–51] also did not stimulate human CRP expression in CRP transgenic mice [42]. 

The specific role of MIF in the regulation of T2D risk factors (e.g., the acute phase reactants CRP, SAA, fibrinogen) has yet to be systemically analyzed. Burger-Kentischer and coworkers showed that immunoneutralization of MIF lowers plasma fibrinogen and IL-6 levels and reduces the expression level of CCAAT-enhancer-binding protein beta (C/EBP*β*) [52] and a similar effect on C/EBP*β* was reported by others [38]. C/EBP*β* is a positive regulator of IL-6, fibrinogen, SAA, and CRP but also a transcription factor for the adhesion molecules VCAM-1 and ICAM-1 [45, 53], and MIF is coexpressed with C/EBP*β*-regulated proteases (e.g., MMP-9) [54] that have been associated with hyperexpression of IL-6 and IL-8 and the development of renal and cardiovascular complications [55, 56]. 

Finally, the identification of prevalent, functional polymorphisms in MIF, which show significant population stratification [20], prompts closer examination of the contribution of the *MIF* locus as a risk factor for T2D, obesity, and cardiovascular disease.

## Figures and Tables

**Table 1:** Relationship between plasma MIF concentrations and body mass index in human studies. Studies are listed on basis of increasing body mass index (BMI). The plasma MIF concentration is provided together with the number of male (m) and female (f) participants and the major effects observed. NS: not specified.

Average BMI	Gender (m; f)	MIF conc (ng/mL)	Effect observed	Reference
22.6 versus 37.5	19; 21	1.2 versus 2.8	Positive correlation of BMI with plasma MIF conc. and MIF mRNA of MNC	[10]

22.6 versus 40.0	16; 16	1.3 versus 3.3	No correlation of plasma MIF with BMI and FFA; pos. correlation with HOMA	[11]

18–25 versus 30–48	0; 46	0.5 versus 1.9	Positive correlation with BMI	[12]

43.0	23; 48	8.4	MIF decreased with weight loss MIF conc. elevated in women with HRT	[13]

32.5 to 30.6	0; 31	16.0 to 5.4	MIF conc. decreased with weight loss	[14]

46.7	5; 22	0.2	MIF conc. increased with weight loss	[15]

27 versus 37	females	NS	MIF production of adipose tissue is positively correlated with BMI	[22]

36	34	NS	mRNA conc positively associated with adipocyte diameter	[23]

**Table 2:** Relationship between plasma MIF concentrations, glucose intolerance, and T2D. The table provides the type of subjects (study groups), the number of subjects per study group, and the plasma/serum MIF concentration together with the major effects. IGT: impaired glucose tolerance; PDR: proliferative diabetic retinopathy; ND: not determined.

Study groups	*n*	MIF conc (ng/mL)	Effect observed	Reference
Control	79	5.2	MIF is elevated in T2D subjects	[24]
T2D	79	20.7		

Caucasians	24	<5.0 in 100%	Fasting MIF conc. are higher in Pima Indians	[25]
Pima Indians	28	>5.0 in 39%		

Normoglycemic	244	4.97	Positive association of MIF with IGT and T2D independent of CRP and IL-6	[26]
IGT	242	7.95		
T2D	236	10.96		

Noncases controls	1632	17.7	Females with* MIF* genotype rs1007888CC have increased risk of T2D	[16]
Future T2D	502	18.5		

Control	257	5.8	Identification of MIF as a novel immune marker to predict progression from IGT to T2D	[27]
Lifestyle intervention	265	6.2		

Control	6	ND	Increased kidney CD74 expression	[28]
Diabetic nephropathy	20			

Control	39	1.8	Increased MIF levels in vitreous of patients with PDR	[29]
PDR	32	11.9		

Nondiabetics	24	1.1	Increased MIF levels in patients with retinopathy	[30]
PDR	40	6.3		

Control diabetics	140	4.3	Higher MIF levels in diabetics with ulcer	[31]
Diabetics with ulcer	170	7.7		
